# A comparison of wetland characteristics between Agricultural Conservation Easement Program and public lands wetlands in West Virginia, USA

**DOI:** 10.1002/ece3.6118

**Published:** 2020-02-20

**Authors:** Katharine E. Lewis, Christopher T. Rota, James T. Anderson

**Affiliations:** ^1^ Davis College of Agriculture, Natural Resources, and Design West Virginia University Morgantown WV USA

**Keywords:** Agricultural Conservation Easement Program, Appalachians, wetland restoration, wetlands

## Abstract

In West Virginia, USA, there are 24 conservation easement program wetlands enrolled in the Agricultural Conservation Easement Program (ACEP). These wetlands are located on private agricultural land and are passively managed. Due to their location within fragmented agricultural areas, wetlands enrolled in ACEP in West Virginia have the potential to add wetland ecosystem services in areas that are lacking these features. We evaluated ACEP wetlands compared to reference wetlands on public land in West Virginia by using surrounding land cover, vegetative cover, and wetland features and stressors such as the presence or absence of erosion, upland inclusion, algal mats, and evidence of impacts from the surrounding landscape as surrogate measurements of wetland function on 13 ACEP wetlands and 10 reference wetlands. ACEP wetlands had higher percentages of tree coverage and a higher proportion of agricultural land in the areas immediately surrounding the wetland. Reference wetlands had higher percent coverage of emergent vegetation and had a higher proportion of forest in the immediate landscape. Our findings suggest that ACEP wetlands provide valuable early successional and forested wetland cover in a state that is largely forested. Because of this, it is important to maintain and even expand ACEP in West Virginia to continue providing a valuable source of early successional wetland habitat.

## Graphical abstract

We studied and compared wetland characteristics on Agricultural Conservation Easement Program (ACEP) wetlands and wetlands located on public land in West Virginia, USA. ACEP wetlands contribute to the diversity of wetland habitat in the state.

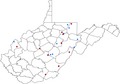

## 1 INTRODUCTION

Wetlands provide numerous ecosystem services and functions including carbon sequestration (Zedler & Kercher, 2005), water filtration (Fennessy & Craft, 2011), nutrient retention (Hansson, Bronmark, Anders Nilsson, & Abjornsson, 2005), flood and storm water storage (Clarkson, Ausseil, & Gerbeaux, 2013), and wildlife habitat (Dahl, 2011). In the United States, wetlands are often exposed to agricultural runoff and impacts from livestock grazing (Mitsch & Hernandez, 2013). Due to the crucial services and functions provided by wetlands, conservation programs and policies have been developed to mitigate historic losses (Gleason & Tangen, 2008a). During European settlement of the conterminous United States, wetlands were often drained for agriculture (Dahl & Allord, 1996). This trend continued well into the twentieth century, with government‐sanctioned wetland drainages across the United States. Agricultural land use accounted for the majority of wetland losses (Frayer, Monahan, Bowden, & Graybill, 1983). In response to historical wetland drainage and degradation, wetland restoration and conservation efforts in the United States included the Swampbuster provision in the Food Security Act of 1985, which ultimately became the Wetland Reserve Program (WRP) in 1990. The WRP allowed agricultural producers to restore or set‐aside wetlands in 30‐year or permanent easements (Votteler & Muir, 2002). In 2014, the Wetland Reserve Program was amended to the Wetland Reserve Easement component of the Agricultural Conservation Easement Program (ACEP) to reflect the program's focus on the conservation of agricultural lands. ACEP is administered under the Natural Resources Conservation Service (NRCS) and continues to provide a mechanism to conserve wetlands on private agricultural lands.

ACEP is a voluntary federal program that works to conserve wetlands and grasslands in working agricultural landscapes. Restoration activities are site‐specific, but often include restoring hydrologic characteristics that existed prior to land manipulation and disturbance. Impacts to the watershed at large are also considered, and structural changes to the land can be used to recreate original hydrologic characteristics. Restoration can also include establishing a wetland plant community or allowing existing seed banks to revegetate the area. Additionally, upland areas can be converted to wetland habitat, or included in the wetland easement if those acres contribute to the functioning of the wetland (Natural Resources Conservation Service, 2017). The ACEP wetlands are typically restored to reflect historic hydrologic regimes, plant communities, and to require little active management after initial restoration. Since its inception in 1996, the Wetland Reserve Program (WRP) and ACEP has established >800,000 ha, or 2 million acres, of restored wetland habitat nationwide (U.S. Department of Agriculture, 2018). In 2016, the NRCS spent approximately $345 million on technical and financial assistance for landowners restoring wetlands through ACEP (U.S. Department of Agriculture, 2018).

There are many benefits to restoring wetlands on private agricultural land including positive impacts on water storage and plant and wildlife biodiversity (Benson, Carberry, & Langen, 2018; Gleason, Euliss, Tangen, Laubhan, & Browne, 2011). Wetlands enrolled in ACEP have the potential to intercept and store floodwater in agricultural areas that have a high volume of runoff (Gleason & Tangen, 2008a). Similarly, converting agricultural areas such as cropland to wetland conservation easements was estimated to reduce soil erosion rates and therefore reduce sedimentation (Gleason & Tangen, 2008b). Given the national scope of ACEP and the cost to the federal government, it is important to evaluate characteristics of enrolled wetlands and ensure they are an adequate complement to existing, naturally occurring wetlands in the same regions. Measuring wetland characteristics allows for an evaluation of the types of wetlands that exist as ACEP easements and can potentially identify the presence of stressors that may inhibit intended wetland benefits.

The land cover and use of surrounding landscapes can have an impact on wetland characteristics through associated stressors or features. It is possible that agricultural areas around wetlands restored through ACEP could influence the vegetative community and introduce different sources of pollution or habitat disturbance, leading to differences in wetland characteristics between ACEP wetlands and wetlands in non‐agricultural landscapes. For example, wetlands that are immediately adjacent to agricultural and livestock areas can be impacted by sedimentation and fertilizer runoff, which can manifest as presence of sediment‐tolerant vegetation (Martin & Hartman, 1987; Poesen, Vandaele, Van, & Wesemael, 1996), algal mats (Lundy, Spencer, Kessel, Hill, & Linquist, 2011), or actual chemical or organic waste in the wetland. Watersheds containing higher proportions of cropland had greater concentrations of nitrogen from fertilizer use than those with lower proportions of cropland (Jordan, Correll, & Weller, 1997), and wetlands directly adjacent to crop or pasture land are prone to waste and fertilizer runoff (Knight, Payne, Borer, Clarke, & Pries, 2000). Measurements of biological integrity based on plant species composition was lower on wetlands adjacent to agricultural lands relative to other land‐cover types, which could be attributed to agricultural runoff and pollution (Stapanian, Gara, & Schumacher, 2018).

Hydrologic characteristics of wetlands are another important indicator of wetland function and their ability to provide ecosystem services. In particular, several characteristics can be used to indicate potential problems in wetland hydrology. Wetlands that have inconsistent fluctuations in hydrology can show evidence of soil cracks or fissures (Rojas, Arzate, & Arroyo, 2002), entrenched streams, and widening or deepening of water upstream (Veselka & Anderson, 2011). Disturbance from human impacts such as construction or the creation of drainage pipes, dams, or culverts can also interrupt hydrology and create unnatural drainage patterns or water fluctuations in wetlands or their associated streams (Lenhart, Veery, Brooks, & Magner, 2011). Hydrophytic vegetation that is dying or rooted vascular vegetation that is submerged can also be caused by unnatural water fluctuations that may indicate that the wetland is not accommodating the water or lack thereof in its system.

The process of wetland creation or restoration can influence vegetative communities. In some cases, created or restored wetlands can have similar plant species composition to naturally occurring wetlands that are managed in the same way but are at younger successional stages than established natural wetlands (De Steven & Gramling, 2013; Evans‐Peters, Dugger, & Petrie, 2012). Similarly, others have found that created wetlands have a greater number of plant species than older, natural wetlands (Confer & Niering, 1992), perhaps due to the earlier successional stage and lack of interspecific vegetative competition in created wetlands. Differences in vegetative structure between mitigated and reference wetlands can vary, though. For example, a comparison between wetlands created through mitigation and naturally occurring reference wetlands in West Virginia found no difference in average coverage of plant species, but created wetlands had higher plant diversity than reference sites (Balcombe et al., 2005). In contrast, Campbell, Cole, and Brooks (2002) found higher plant species richness and vegetative coverage on naturally occurring wetlands relative to created wetlands in Pennsylvania. In the Prairie Pothole Region, wetland restoration through WRP increased native plant species richness when compared with agricultural areas that did not have a restored conservation easement (Laubhan & Gleason, 2008). In New York, restored WRP and Partner's for Fish and Wildlife Program wetlands had similar species richness and vegetative forage quality as reference wetlands (Benson, Carberry, & Langen, 2019). Another way wetland creation or restoration can impact vegetative communities is through the presence of invasive vegetative species. Restored wetlands could be more susceptible to colonization by invasive clonal graminoids that are difficult to eradicate (Rojas & Zedler, 2015), due to the lack of established vegetation at the start of restoration. Differences in vegetative characteristics and successional stage between created or restored wetlands and naturally occurring wetlands may provide diverse wildlife habitat that complements naturally occurring wetlands. Differences between these types of wetlands provide a mosaic of diverse wetland habitats. It is important to note these characteristics for restored wetlands, and to compare them to the characteristics of naturally occurring wetlands to determine whether easement wetlands are contributing habitat and are functioning unimpeded from stressors from the surrounding environment.

For this study, we compared characteristics of ACEP wetlands on agricultural lands with nearby reference wetlands on public lands in West Virginia. While West Virginia is primarily a forested state with <1% of its area covered by wetlands (Fretwell, Williams, & Redman, 1996), the wetlands located in the state provide important ecosystem services and habitat precisely because of their scarcity. Wetland losses in West Virginia have been primarily due to agricultural land development, along with other forms of human development (Dahl & Allord, 1996). Therefore, programs such as ACEP that restore wetlands on agricultural lands is an important tool in regaining previously lost wetland cover. Previous research evaluating specific wetland characteristics in West Virginia compared naturally occurring wetlands with wetlands created through mitigation or occurred on only a small subsample of ACEP sites (Balcombe, Anderson, Fortney, & Kordek, 2005a, 2005b; Balcombe et al., 2005; Clipp, Peters, & Anderson, 2017; Strain, Turk, & Anderson, 2014). Additionally, past wetland research compared actively and passively managed wetlands (Anderson & Smith, 1998, 2000; Fleming et al., 2015; Kaminski, Baldassarre, & Pearse, 2006; O'Neal, Heske, & Strafford, 2008). To our knowledge, a comparison of wetland features on passively managed conservation easements relative to reference wetlands has not been completed. Such a comparison will allow us to determine how ACEP wetlands differ from reference wetlands and could provide valuable insight to landowners and managers that maintain wetlands in the state by identifying characteristics as well as potential sources of stressors. Our objectives for this study were to conduct a state‐wide comparison of wetland characteristics on ACEP wetlands located on private land with a set of reference wetlands on public land to: (a) evaluate the characteristics of wetland ecosystems restored on agricultural land and (b) determine how surrounding land use, hydrologic and physical characteristics, and vegetative composition on ACEP wetlands compare to other available wetland habitat within West Virginia.

## 2 MATERIALS AND METHODS

### 2.1 Site Selection

**Figure 1:**
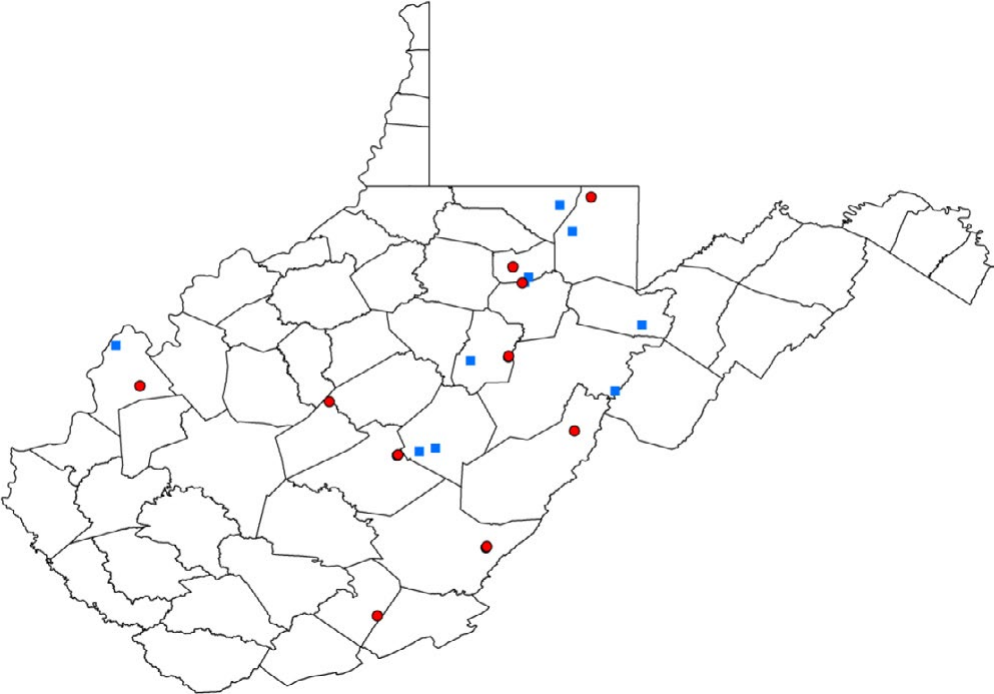
Wetlands enrolled in the Agricultural Conservation Easement Program (ACEP) with the exception of those located in the Eastern Panhandle of the state, administered through the Natural Resources Conservation Service in West Virginia, USA, along with reference wetlands located on public land on wildlife management areas, state parks, and the Nature Conservancy land. Blue squares represent reference sites, and red circles represent ACEP wetland sites

We conducted assessments of ACEP and reference wetland characteristics in West Virginia in May of 2017. This study occurred on 13 ACEP and 10 reference wetlands located in the Allegheny Mountain and Appalachian Plateau physiographic provinces of West Virginia (Figure 1). West Virginia is a predominantly mountainous, forested state: approximately 80% of West Virginia is forested (Morin, Domke, & Walters, 2017), while <1% of the state's surface is covered by wetlands (Tiner et al., 1994). In West Virginia, there are 24 wetland easements enrolled in ACEP. We were denied access to five sites by the landowners and excluded the six ACEP wetlands located in the Eastern Panhandle of the state due to a lack of available reference wetlands in that region, and the fact that the Eastern Panhandle consists of the Valley and Ridge and Great Valley physiographic provinces, which generally differ between the Allegheny Mountain and Appalachian Plateau provinces (West Virginia Geological & Economic Survey, 2017).

The restoration process for the ACEP wetlands began between 1996 and 2012 (Table S1). Wetland sites were located on private agricultural land with the exception of one site located on a Wildlife Management Area within an agricultural landscape. Most ACEP wetlands were adjacent to pasture, with one site being surrounded by row crops of corn (*Zea mays*). Conservation practices implemented on ACEP wetlands varied, but generally consisted of erecting livestock exclusion fencing, creating alternative water sources or reinforcing substrate with gravel at water access points, restoring hydrology by removing tile drains, plugging drainage ditches, or excavating small pools, and occasional plantings. Wetlands ranged in size from <0.91 ha to 32 ha, with an average size of 9.9 ha (variance = 125.2, Table S1) and were classed as either palustrine emergent, forested, or scrub–shrub wetlands (Cowardin, Carter, Golet, & LaRoe, 1979). Emergent wetlands were dominated by rooted hydrophytic vegetation such as cattails (*Typha* spp.), bulrush (*Scirpus* spp.), and sedges (*Carex* spp.). Forested wetlands had an overstory of trees and were dominated by woody vegetation > 6 m tall such as American sycamore (*Platanus occidentalis),* pin oak (*Quercus palustris*), and black willow (*Salix nigra*). Scrub–shrub wetlands were dominated by woody vegetation < 6 m tall such as alders (*Alnus* spp.) and buttonbush (*Cephalanthus occidentalis*; Cowardin et al., 1979). ACEP wetland boundaries were defined by the easement agreement boundary and were often demarcated by a fence. In some cases, these easement boundaries included areas of adjacent upland habitat that contributed to the quality of the wetland, and also often included different wetland types (e.g., forested, emergent, shrub–scrub) within one boundary.

We compared characteristics of ACEP wetlands with reference wetlands located on public land. We used the National Wetlands Inventory (NWI) data layer (U. S. Fish & Wildlife Service, 2016) to identify wetlands in WV wildlife management areas, WV state parks, WV state forests, and property owned by the Nature Conservancy. We limited this list to include only wetlands that were ≤32 ha to be consistent with the sizes of ACEP wetlands (Table S1) and classified as emergent, scrub–shrub, or forested to reflect the categories of ACEP wetlands and provide a more direct comparison. Finally, to minimize other potentially confounding factors between ACEP and reference wetlands, we constrained reference wetlands to the same or adjacent counties as ACEP wetlands. Due to the lack of wetland land cover in the state, only 13 available reference wetlands met our criteria. These sites were predominantly surrounded by forest patches that consisted of mixed deciduous tree species. Reference wetlands were delineated by the National Wetland Inventory by wetland type according to the Cowardin et al. (1979) classification system. Because of this, reference wetland boundaries did not contain more than one wetland type and had the potential to be surrounded by other wetland types in a complex. We conducted our assessments on 10 reference wetlands randomly selected from a group of 13 potential reference sites with an average size of 5.75 ha (variance = 16.3, Table S1). Because we were constrained by time and conducted our characterizations during the month of May in 2017, we were limited in the number of sites we were able to visit and assess.

We assessed characteristics of wetland sites through the use of presence or absence of different stressors or features that were used to describe the wetland hydrology and the potential impacts from surrounding land use. We also measured ground cover and vegetative features of the wetlands on transects in percentage cover categories. These ground‐cover transects also provided insights into the presence of features or stressors as we measured the general vegetative composition of each wetland, the percent cover of invasive species and unvegetated mudflats, and the amount of open water on each transect which pertains to the hydrologic features of the wetland.

### 2.2 Wetland characteristics assessments

We measured wetland characteristics based on the West Virginia Wetland Rapid Assessment Procedure training manual (Veselka & Anderson, 2011). Data collection protocols described within this manual were designed to measure indications of wetland function, using vegetative data and the presence or absence of different features as surrogate measurements for function. These assessments were designed to be completed in a single visit and be robust to seasonal change (Veselka & Anderson, 2011). Our assessments were used to evaluate the land use immediately surrounding wetlands, vegetative characteristics within wetlands, wetland hydrology, and the presence or absence of a set of wetland features and stressors. We performed supplementary verification on hydrologic regimes by making additional site visits during the winter and summer for concurrent studies of avian occupancy (Lewis, Rota, Lituma, & Anderson, 2019) and turtle communities (Gulette, Anderson, & Brown, 2019).

**Figure 2:**
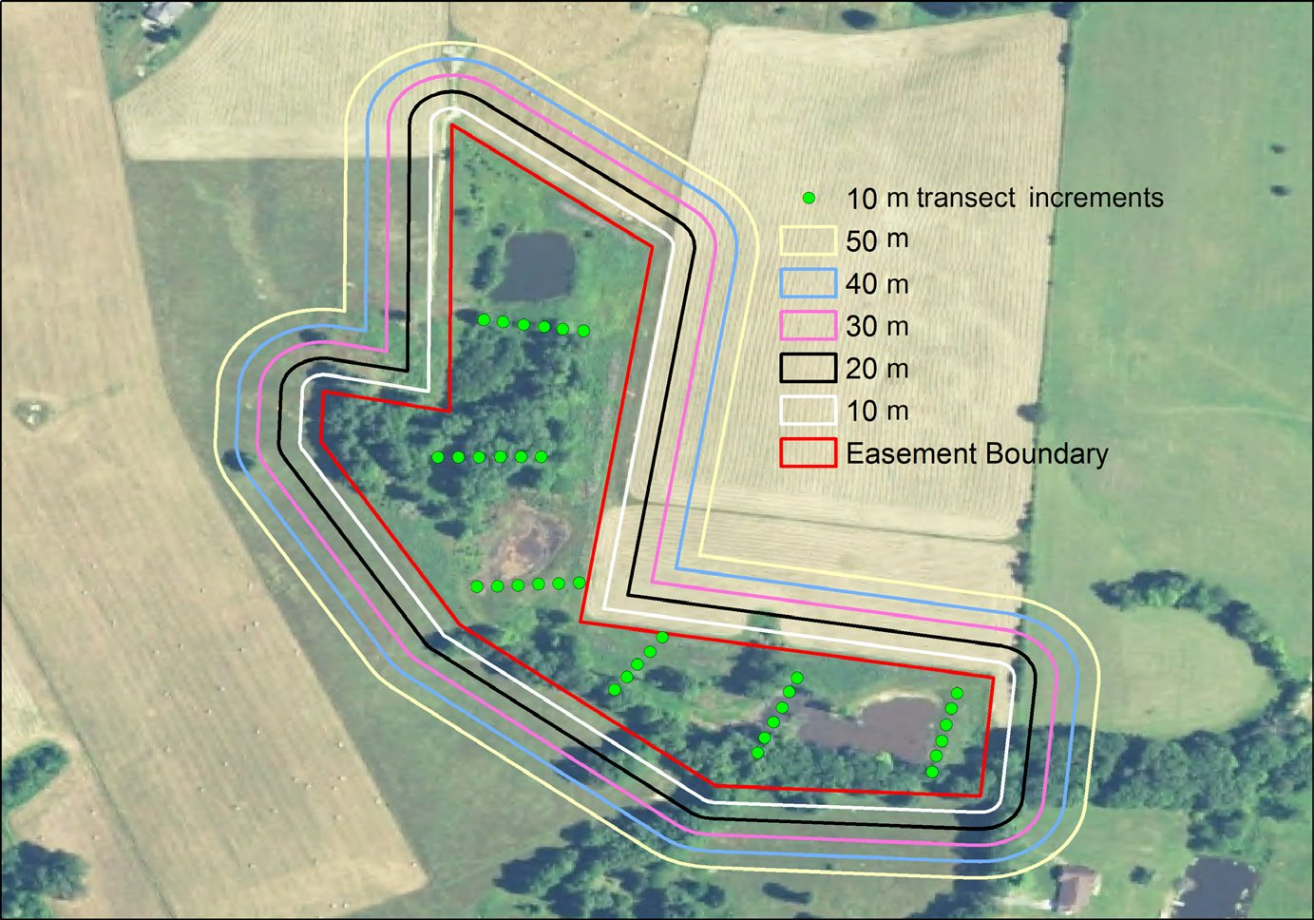
Example of a 50 m buffer separated by 10 m buffer increments, and vegetative transects around an Agricultural Conservation Easement Program wetland site in West Virginia, USA. Red represents the wetland easement boundary. Transects are placed every 78 m, are 50 m long and are broken into 10 m increments represented by green dots

We evaluated land use surrounding wetlands by characterizing land‐cover types within 50 m of each wetland boundary (Veselka & Anderson, 2011) on 12 out of the 13 ACEP sites. We were unable to assess the land cover surrounding one ACEP site due to high water levels restricting our access. We classified dominant land cover into 6 categories describing the principle use of the land (Table 1). We determined dominant land‐cover type by first placing 5 transects, spaced 10 m apart, around the wetland perimeter (Figure 2). We then walked the perimeter of each wetland site and visually characterized the dominant land cover within each of the 5 transects (Veselka & Anderson, 2011).

**Table 1:** List of land‐cover categories used to categorize the surrounding area around Agricultural Conservation Easement Program (ACEP) and reference wetland sites in West Virginia in May of 2017 within a 50 m buffer and descriptions of land‐cover categories

Land‐cover buffer category	Description
Forested	Dominated by tree stands, >50% tree coverage
Wetland	Standing water or other wetland types (e.g., scrub shrub, emergent, forested) that extends beyond the ACEP easement boundary or the reference wetland polygon.
Roads	1 or 2 lane paved roads, low‐use recreational roads such as gravel paths
Agriculture	Mowed fields or fields used by livestock; Dairy farm operations that include cattle feed lots, impervious surfaces such as milking parlors, and unvegetated cattle enclosures
Residential	Single family homes, apartments, townhouses

After characterizing land use surrounding the wetlands, we recorded the presence or absence of several wetland features and stressors (Table 2). We evaluated these wetland features and stressors on 50 m transects that we placed perpendicular to the flow of water within each wetland (Veselka & Anderson, 2011; Figure 2). We included one transect per 0.6 ha, spaced 78 m apart. All wetlands, regardless of size, had at least one transect (Veselka & Anderson, 2011). If a wetland feature or stressor was recorded on any transect, we considered it present on the entire wetland site. We further measured the percent cover of 23 different categories of vegetative or ground cover within the wetlands on each transect. Included in these measurements were woody vegetation, forb or grass cover, invasive species, open water, aquatic vegetation, mosses and lichen, and rock cover (Table 3). We measured these characteristics by subdividing the 50 m transects into 10 m increments and characterizing vegetation and ground cover at each increment with a Daubenmire cover‐class category (Daubenmire, 1959).

**Table 2:** Wetland features and stressors assessed on each Agricultural Conservation Easement Program (ACEP) wetland and reference wetland located in West Virginia, USA, in May of 2017

Wetland feature and stressor presence/absence variables	Description of variables (Veselka & Anderson, 2011)	Ratio of ACEP sites present	Ratio of reference sites present
Upland inclusion	Upland vegetation/lack of hydrology present	13/13	10/10
Stream channel	Any stream flowing through the wetland	10/13	8/10
Entrenched streams	A stream channel that is not connected to the surrounding wetland and has eroding banks or slopes	7/13	6/10
Permanent flooding	Surface water appears to be present throughout the entire year	8/13	8/10
Seasonal flooding	Surface water is present only during a portion of the year	11/13	1/10
Saturated soil	Soil is saturated to the surface, but not flooded	12/13	10/10
Erosion	Stream banks or slopes displaying sloughing indicative of erosion	5/13	2/10
Construction	Earth‐moving or construction activity	1/13	0/10
Sediment‐tolerant vegetation	Species indicative of sedimentation such as cattails (*Typha* spp.)	0/13	0/10
Impervious surface runoff	Runoff from impervious surfaces such as roads	2/13	0/10
Agricultural effects	Presence of fertilizers, manure spreading operations, livestock present	4/13	0/10
Algal mats	Clumps or mats of green, opaque, filamentous algae	2/13	1/10
Organic waste	Piles of grass clippings, woody debris, or other organic matter	0/13	0/10
Spill/ Odor	Odors or spills indicating pollution from agricultural or chemical sources	4/13	1/10
Vegetated mounds	Soil mounds indicating digging or construction that have vegetation	6/13	6/10
Coarse woody debris	Dead woody vegetation such as logs or stumps	8/13	8/10
Snags	Dead woody vegetation that is upright and >6 meters tall	8/13	6/10
No surface water inlet/outlet	Indicates isolated wetland that is artificially flooded	0/13	0/10
Relatively non‐permanent waterway	A channel or stream that is not consistent and only occurs after precipitation or flooding	0/13	1/10
Dams	Evidence of beaver dams	2/13	0/10
Water control structures	Spillway or dam that controls the flow of water into and out of the wetland	5/13	1/10
Ditch	Man‐made channel that consistently conveys water to the wetland	0/13	0/10
Perched culvert	Culverts with one or both ends at an elevation different than the water	0/13	0/10
Tile	Underground drainage pipe in fields to drain water	0/13	0/10
Dike/levee	Man‐made berm that acts as the border between upland and wetland	0/13	0/10
Upstream widening of wetland	Indication of impacts from impounded water	0/13	0/10
Upstream deepening of wetland	Indication of impacts from impounded water	0/13	0/10
Railroad tracks	Adjacent railroad tracks that may impound drainage	1/13	2/10
Rotten egg smell		1/13	1/10
Dead/dying vegetation due to hydrology	Water stressed vegetation that is identified by water levels	1/13	0/10
Filamentous algae	Algae that can occur in algal mats and could indicate eutrophication from agricultural runoff	1/13	2/10
Submerged rooted vascular vegetation	An indication that recent flooding has occurred through rooted vegetation submerged under the water or partially sticking up out of the water	2/13	0/10
Exposure of submerged roots	Exposure of the roots of vegetation that would usually be submerged under water that is aquatic plants	2/13	0/10
Mines	Previously mined lands indicated by strips of unvegetated areas on forested hills	1/13	0/10
Soil cracks	Cracks or fissures in the soil indicating water fluctuations and periods of drying	2/13	0/10

**Table 3:** Vegetative cover classes and descriptions of cover classes measured on transects conducted on Agricultural Conservation Easement Program (ACEP) and reference wetlands located in West Virginia, USA, in May of 2017 (Cowardin et al., 1979; Veselka & Anderson, 2011)

Transect vegetation cover classes	Description of cover classes
Rock	Bare rock
Unvegetated mud flat	Areas along the shoreline of water features, areas of wet soil that do not have any plant growth
Open water	Ponds, streams, or areas where water is deep enough to obscure any vegetation
Emergent vegetation	Rooted hydrophytic vegetation such as *Typha*, *Carex* spp.
Moss/ lichen	Mosses or lichens
Submerged aquatic vegetation	Rooted vascular vegetation completely submerged in water such as *Potamogeton* spp.
Shrub: Broad‐leaved deciduous	Shrubs that lose leaves yearly such as buttonbush (*Cephalanthus occidentalis)*
Shrub: Needle‐leaved deciduous	Needle‐leaved shrubs or trees < 6 meters tall that lose their needles yearly including larch (*Larix* spp.) or bald cypress (*Taxodium distichum*)
Shrub: Broad‐leaved evergreen	Broad‐leaved shrubs that retain their leaves throughout the year such as mountain laurel (*Kalmia latifolia*)
Shrub: Needle‐leaved evergreen	Needle‐leaved shrubs and trees <6 meters tall that retain their needles throughout the year such as pine shrubs (*Pinus* spp.) and fir shrubs (*Abies* spp.)
Shrub: Dead	Dead shrub
Tree Canopy: Broad‐leaved deciduous	Broad‐leaved trees that lose their leaves yearly such as oaks (*Quercus* spp.) and maples (*Acer* spp.)
Tree Canopy: Needle‐leaved deciduous	Needle‐leaved trees that lose their needles yearly including larch (*Larix* spp.) or bald cypress (*Taxodium distichum*)
Tree Canopy: Broad‐leaved evergreen	Broad‐leaved trees that retain their leaves throughout the year such as *Magnolia* spp.
Tree Canopy: Needle‐leaved evergreen	Needle‐leaved trees that retain their needles throughout the year such as pines (*Pinus* spp.) and firs (*Abies* spp.)
Tree: Dead	Dead trees
Invasive herbaceous	Non‐native forb species such as purple loosestrife (*Lythrum salicaria*) and Japanese knotweed (*Polygonum cuspidatum*)
Invasive aquatic	Non‐native aquatic alga and plants such as hydrilla (*Hydrilla verticillata*) and Eurasian watermilfoil (*Myriophyllum spicatum*)
Invasive grass	Invasive grass species such as reed canary grass (*Phalaris arundinacea*) and Phragmites (*Phragmites australis*)
Invasive shrub	Non‐native shrub (<6 meters tall) such as multiflora rose (*Rosa multiflora*), honey‐suckle bush (*Lonicera* spp.)
Invasive tree	Non‐native tree (>6 meters tall) such as autumn olive (*Elaeagnus umbellate*) and tree of heaven (*Ailanthus altissima*)
Nutrient‐/sediment‐tolerant species	Invasive plant species that indicate excess sediment or nutrient inputs due to their ability to survive in such conditions, that is, Japanese stiltgrass (*Microstegium vimineum)* and jointed grass (*Anthrazon hispidus*)

We classified these data into surrounding land use, hydrologic and physical characteristics, and vegetative composition categories. We categorized potential stressors stemming from surrounding land use to include invasive species cover and the presence of sediment‐ and nutrient‐tolerant species, algal mats, vegetated mounds, evidence of construction, direct discharge, organic waste, spills or odors, and filamentous algae. The indicators of hydrologic characteristics included variables such as open water that we measured on transects and the presence or absence of streams, soil saturation, flooding regimes, erosion, exposure of usually submerged roots, dead vegetation due to hydrology, submerged rooted vascular vegetation, soil cracks, human‐made water control structures, upstream widening or deepening of streams, water outlets but no inputs, and flowing drainage ditches leaving the wetland. Other features such as the presence of snags or coarse woody debris contribute to the structural make‐up of the wetlands, which align with the vegetative characteristics we measured on the transects in 10 m increments.

### 2.3 Statistical analyses

We compared land‐cover classifications between ACEP and reference site buffers using multinomial regression (McCullagh & Nelder, 1989). Our response variable was the count of each land‐use classification from the 5 transects surrounding each wetland, and our predictor variable was wetland type: either ACEP or reference. We fit multinomial models with the “multinom()” function in package “nnet,” version 7.3‐12 (Ripley & Venables, 2016), within program R version 3.3.1 (R Core Team, 2016). We additionally calculated contrasts in the probability of observing each land‐use category between ACEP and reference wetlands, while adjusting *p*‐values for multiple comparisons, with the “emmeans()” function in package “emmeans” version 1.1.3 (Lenth, Love, & Herve, 2018).

We compared the probability that a wetland feature or stressor was present at ACEP or reference wetlands with logistic regression at the wetland site scale using the “glm()” function in package “stats” version 3.3.1 (R Core Team, 2016). Our response was the presence or absence of the wetland stressors or features, and our predictors were the ACEP or reference wetland type.

We compared vegetative characteristics between ACEP and reference sites using ordinal logistic regression (Gelman & Hill, 2007). Our response variable for this analysis was the Daubenmire cover‐class category recorded for each vegetative characteristic. We therefore selected ordinal logistic regression because this analytical approach is appropriate when response variables are categorical and follow a natural ordering (Gelman & Hill, 2007). We fit ordinal logistic regression models with the “polr()” function in package “MASS” (Ripley & Venables, 2018) within Program R version 3.3.1 (R Core Team, 2016).

## 3 RESULTS

**Figure 3:**
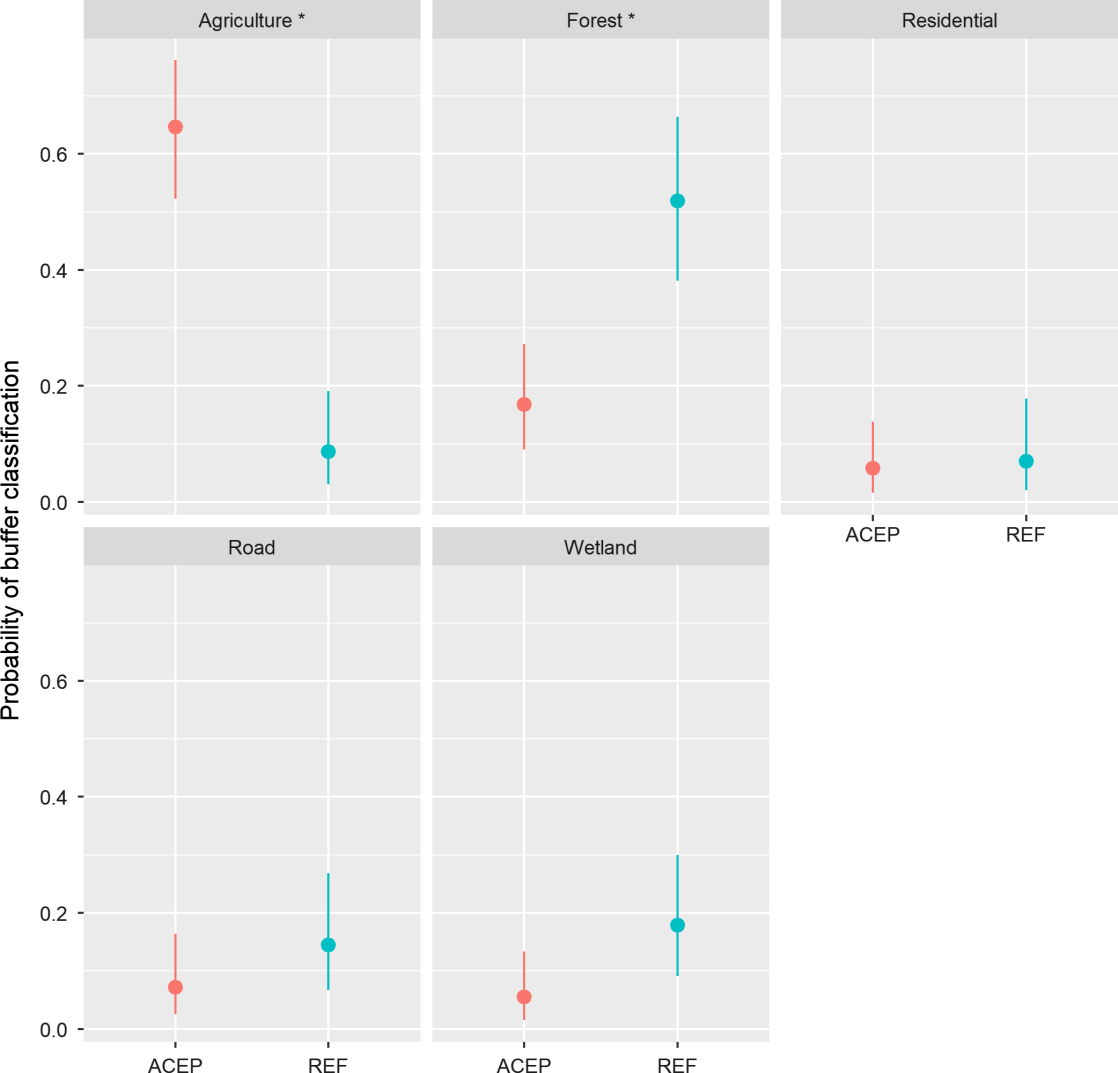
Probability of land‐use buffer classification in the surrounding 50 m around Agricultural Conservation Easement Program wetland sites and reference sites located on public land on wildlife management areas, state parks, and the Nature Conservancy land in West Virginia. Land‐use data were collected in May of 2017 using visual characterization of the dominant land cover within a 50 m buffer around study sites. Dots represent point estimates and vertical lines are the 95% confidence intervals

We assessed dominant land cover surrounding wetlands at 12 ACEP and 10 reference sites in a 50 m buffer immediately around each wetland (Veselka & Anderson, 2011). We found that ACEP and reference sites differed in the dominant land cover surrounding the wetlands. The land cover immediately adjacent to ACEP sites was significantly more likely to be classified as agriculture relative to reference sites (log odds ratio [LOR] = 2.37, *SE* = 0.49, *p* < .01; Figure 3), and the land cover immediately adjacent to reference sites was significantly more likely to be classified as forest (LOR = −0.92, *SE* = 0.40, *p* < .05; Figure 3). The probability of classifying land use immediately outside wetland boundaries as wetland, residential, or road did not differ between ACEP and reference sites (*p* > .05; Figure 3).

**Figure 4:**
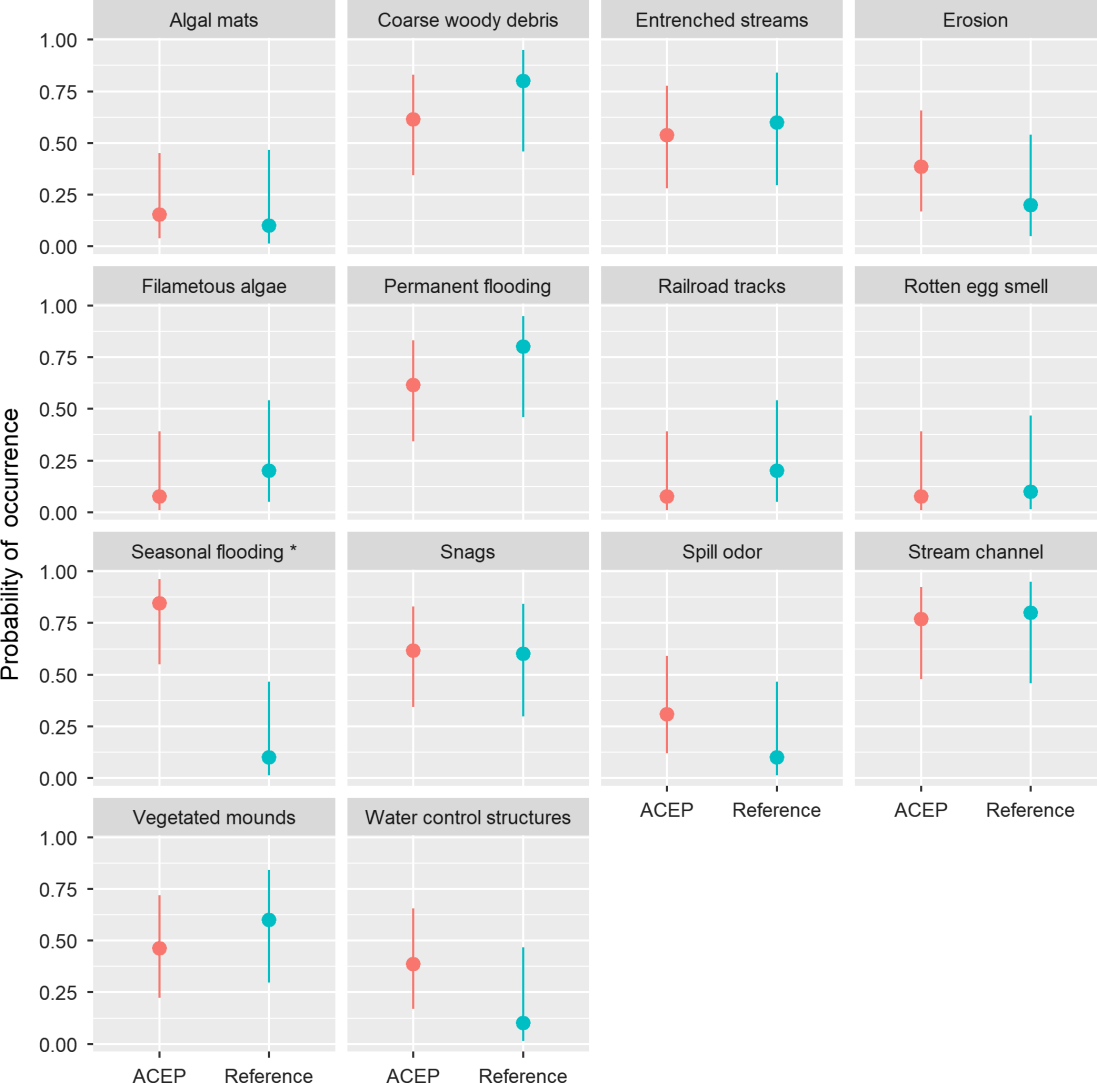
Results of logistic regression conducted with wetland features or stressors as the response variable and Agricultural Conservation Easement Program (ACEP) wetland or reference wetland on presence/ absence data of wetland features or stressors on ACEP and reference wetland sites located in West Virginia, USA, measured in May 2017. Asterisks represent statistically different probabilities

We recorded the presence or absence of wetland features and stressors at 13 ACEP and 10 reference wetlands. Of the hydrological characteristics that we measured, the probability of seasonal flooding was greater at ACEP wetlands relative to reference wetlands (LOR = −3.90, *SE* = 1.3, *p* < .01; Figure 4). We found no differences in other hydrological features between ACEP and reference sites such as the presence of stream channels, entrenched streams, or permanent flooding (*p* > .05). Other variables such as upland inclusion and saturated soils were present on all sites with the exception of one ACEP site without saturated soil. The presence of construction, sediment‐tolerant vegetation, impervious surface runoff, agricultural effects, organic waste, no surface water inlet or outlet, relatively non‐permanent waterways, ditches, perched culverts, tiles, dikes or levees, upstream widening or deepening of wetland, dead vegetation due to hydrology, submerged rooted vascular vegetation, exposure of submerged roots, mines, and soil cracks were absent from most if not all sites. These variables that were either present on all sites or absent on all precluded analysis.

**Figure 5:**
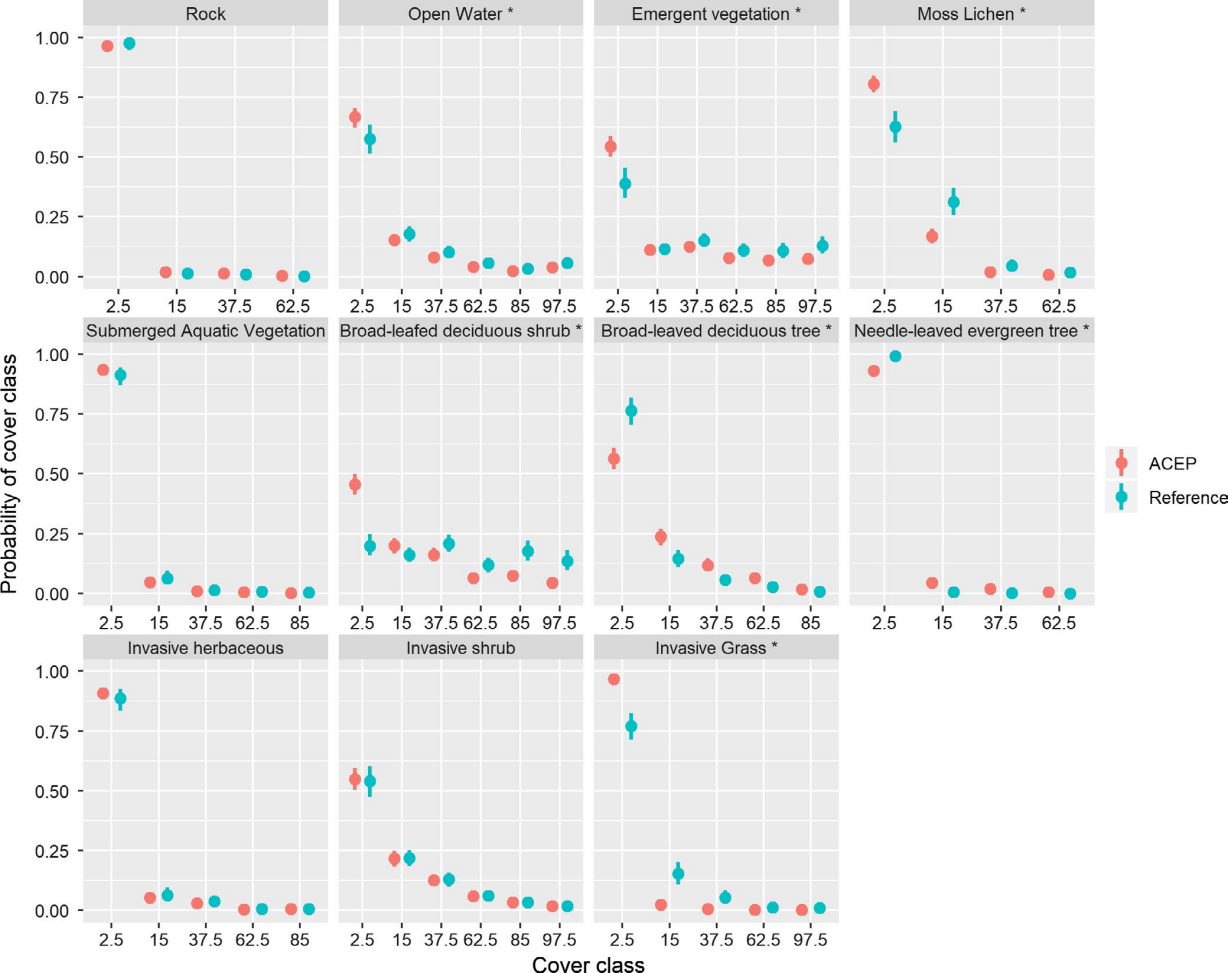
Probability of classifying environmental characteristics into ordinal cover‐class categories at ACEP and reference wetlands taken on transects in Agricultural Conservation Easement Program wetland sites and reference sites located on public land on wildlife management areas, state parks, and the Nature Conservancy land in West Virginia. Data were collected in May 2017. Asterisks represent statistically different probabilities

We recorded vegetative and ground cover along 106 total transects at 13 ACEP wetlands (mean number of 7 transects per wetland, min = 1, max = 21), and along 44 transects on 10 reference wetlands (mean of 4 transects per wetland, min = 1, max = 10). We found that ACEP and reference sites differed in several vegetative or ground‐cover characteristics. At ACEP sites, Daubenmire cover‐class scores were significantly higher for broad‐leaved deciduous trees (LOR = −0.94 *SE* = 0.19, *p* < .01) and needle‐leaved evergreen trees (LOR = −2.7, *SE* = 1.02, *p* < .01; Figure 5). At reference sites, Daubenmire cover‐class scores were significantly greater for emergent vegetation (LOR = 0.63, *SE* = 0.16, *p* < .01), moss and lichen (LOR = 0.91, *SE* = 0.19, *p* < .01), invasive grass (LOR = 2.2, *SE* = 0.31, *p* < .01)*,* and broad‐leaved deciduous shrubs (LOR = 1.2, *SE* = 0.16, *p* < .01; Figure 5). We also found differences in hydrological characteristics, with Daubenmire cover‐class scores for open water significantly greater on reference sites compared to ACEP sites (LOR = 0.39, *SE* = 0.16, *p* < .05). Daubenmire cover‐class scores did not differ significantly between ACEP and reference wetlands between the vegetative characteristic of submerged aquatic vegetation, or the stressors from potential surrounding land use of invasive herbaceous material, or invasive shrub (*p* > .05; Figure 5). We did not observe the unvegetated mudflat category at any of our reference sites. Additionally, certain categories were not found at all or occurred only in low cover‐class scores on both ACEP and reference sites. These categories included the following: needle‐leaved deciduous shrubs, broad‐leaved evergreen shrubs, needle‐leaved evergreen shrubs, dead shrubs, needle‐leaved deciduous trees, broad‐leaved evergreen trees, dead trees, invasive trees, invasive aquatic plants, nutrient‐tolerant species, and sediment‐tolerant species.

## 4 DISCUSSION

We observed differences between ACEP and reference wetlands in landscape setting, vegetation, and hydrological characteristics. By design, ACEP and reference wetlands were largely situated within different landscapes. ACEP wetlands were located within working agricultural lands, while reference wetlands were located on public lands within forested landscapes. This directly led to the observation that ACEP sites had higher proportions of pasture within a 50 m buffer around the wetland edge, while reference wetlands had higher proportions of forest within a 50 m buffer around the wetland edge. Given that ACEP is focused on the conservation of wetlands on agricultural lands, and ACEP wetlands were located directly adjacent to agricultural fields, these findings are not surprising. While not surprising, the differences in surrounding landscape between ACEP and reference wetlands highlight the importance of ACEP in West Virginia. ACEP is a means of contributing wetlands in a variety of landscapes. Most of our reference sites were located within forested areas, as much of West Virginia is forested overall. Through ACEP, wetlands were restored in areas other than forests and contributed to a diverse array of wetland habitat in the landscape.

The differences in surrounding land had the potential to subject ACEP and reference wetlands to different stressors. While we found differences in the surrounding land use of ACEP and reference wetlands, we did not find differences in characteristics that may have indicated impacts from land use such as the presence of algal mats, chemical and agricultural spills or odors, vegetated mounds, water control structures, railroad tracks, or filamentous algae.

Although wetlands situated within agricultural landscapes can be subject to disrupted hydrology, agricultural runoff, and disturbance from livestock (Knight et al., 2000; Lenhart et al., 2011; Lundy et al., 2011; Mitsch & Hernandez, 2013), we found no differences in stressors between ACEP and reference wetlands that could be indicators of such conditions. The presence or absence of filamentous algae and algal mats would indicate the presence of pollution from animal waste or nutrient runoff (Conley et al., 2009). Additionally, sediment‐tolerant and nutrient‐tolerant vegetations, bank erosion, and vegetated mounds could be the result of human or livestock disturbance from fertilizer or sediment runoff, human‐made construction, or livestock physical disturbance. The intensity of farming practices on the surrounding landscape can contribute variable nutrient runoff loads, with higher erosion rates associated with conventional tillage methods over no‐till or conservation tillage methods contributing higher loads of phosphorous and nitrogen to the surrounding watershed (Harmel et al., 2006). Since the ACEP sites included in this study were adjacent to low‐intensity, small‐scale livestock, or row crop operations, the nutrient load in runoff from adjacent fields may not have contributed high amounts of nutrient runoff. Many of the ACEP sites were adjacent to pasture, with small numbers of livestock on the fields only during portions of the year.

The only hydrological characteristics that differed between ACEP and reference wetlands was an increased likelihood of seasonal flooding on ACEP wetlands and a higher percentage of open water on reference wetlands. The restoration process for ACEP involves allowing historical hydrology to return. Wetlands typically have saturated soils, the water table is near the surface, and, or, there is standing water during some period of the year (Cowardin et al., 1979). Therefore, to restore a sustainable wetland, a source of water such as a stream, periodic flooding, or permanent flooding due to depressions is important (U.S. Environmental Protection Agency, 2000). Seasonal flooding on ACEP wetlands indicates that ACEP wetlands are providing floodwater storage for excess water during different parts of the year and that the restored wetlands are returning to and maintaining hydrological regimes that were present before agricultural development. In general, set‐aside conservation practices such as wetland easements function as important floodwater storage areas (Gleason & Tangen, 2008a). Seasonal flooding also provides an important resource for wildlife that make use of ephemeral water such as amphibians or waterbirds that forage in shallow water (Gleason & Tangen, 2008a; Paton & Crouch, 2002).

The higher percentage of open water on reference wetlands indicates that these sites had more permanent standing water features in the form of a large lake or permanent stream that were not present due to flooding. While the presence of seasonal flooding on ACEP wetlands would also contribute to open water on the wetlands, the reference sites had larger expanses of standing open water due to the permanent nature of these water features. Past studies in the Prairie Pothole Region found that conservation easement wetlands were isolated from wetland complexes, and over‐represented the historical number of seasonal wetlands (Galatowitsch & van der Valk, 1996); therefore, the ACEP wetlands in this study may be more likely to have seasonal flooding due to the nature of their location and isolation from wetland complexes, while the reference wetlands had more standing open water that did not fluctuate or exist as only saturated soil like the ACEP wetlands.

The other difference between reference and ACEP sites we observed concerned differences in ground cover and successional stages. Reference sites had more broad‐leaved deciduous shrubs and emergent vegetation, while ACEP sites had more trees overall, specifically broad‐leaved deciduous and needle‐leaved evergreen trees. The vegetative composition of the reference wetlands, specifically a lack of mature woody vegetation, indicates that these sites existed more as early successional wetlands. Early successional habitats, including wetlands, are important sources of biodiversity and provide open, herbaceous habitat for a wide range of taxa that are not associated with mature growth or interior habitats (Askins, 2001; Scharine, Nielsen, Schauber, Rubert, & Crawford, 2011). The more forested portions of wetlands that we observed on ACEP easements provide critical habitat for breeding passerine species (Sallabanks, Walters, & Collazo, 2000), along with mammals and herpetofauna. In a state that is limited in wetland cover, a diversity of existing early successional wetlands on public land and wetlands that contain both forested and early successional herbaceous areas on private wetland easements is important.

A higher incidence of trees on ACEP sites may seem to contradict the higher prevalence of forests in buffers surrounding reference sites. However, landowners enrolling wetlands in ACEP are permitted to enroll upland areas adjacent to wetlands if those areas contribute to or protect the functioning of the wetland (Oaky, 2003). Because of this, ACEP sites had more areas of tree stands within the wetland boundary than the reference sites, which were delineated strictly by wetland types and did not include additional forested areas. Prior to restoration, ACEP wetland easement sites often had altered hydrology that reduced the hydroperiod enough for woody vegetation to become established but not enough for active farming to occur. The lack of tillage and grazing promoted woody vegetative growth. Similarly, ACEP sites contained multiple wetland classifications within their boundary (i.e., forested wetland areas in addition to freshwater emergent or scrub shrub), while reference wetland boundaries generally lacked forested vegetative classes within the boundaries. It is also likely that reference wetlands had hydrological characteristics such as permanent and semi‐permanent water regimes that prevented the growth of tall woody vegetation such as trees, thus keeping them in an emergent vegetative state.

Previous studies that evaluated plant communities in agricultural landscapes reported a lack of differences in terms of vegetative cover between wetlands located on agricultural land and those that existed within other landscape matrices (Confer & Niering, 1992; Gleason & Rooney, 2017; Gleason et al., 2011; Laubhan & Gleason, 2008; Tapp & Webb, 2015). The differences in vegetation we observed between ACEP and reference sites indicates that ACEP wetlands are acting as a complement to other available wetlands throughout the state. The additional vegetative structural diversity afforded by having more tree cover on some wetlands in West Virginia provides diverse, unique habitat for wetland plants when added to naturally occurring wetlands dominated by emergent and shrub vegetation. Because the ACEP wetlands included in this study are located on private agricultural land that was previously pasture or cropland, this program is a source of diverse wetland habitats and associated ecosystem services that would otherwise not be present on the agricultural fields these wetlands are restored on.

In addition to differences in general vegetative structure between site types, we also found a significant difference in the percentage of invasive grasses such as reed canary grass (*Phalaris arundinacea*) between ACEP and reference sites. The higher percentage of invasive grass on reference sites compared to ACEP wetlands is contrary to what others have found. Studies comparing the vegetative community on restored and reference wetlands in New York found no difference in invasive plant community composition between sites (Benson et al., 2019). However, studies in the southeast United States evaluating conservation easement wetland vegetative communities were consistent with our findings that ACEP wetlands have predominantly native wetland vegetation (De Steven & Gramling, 2012, 2013). Wetlands restored through ACEP are frequently planted with native species at the time of restoration (Natural Resources Conservation Service, 2017), so invasive species may not have become as easily established if they were not already present on the site. Moreover, diligence in recreating historic hydrology likely contributed to producing conditions most conducive to native vegetation. Reference wetlands were publicly accessible and were potentially more susceptible to invasive species establishment due to propagule dispersal through unintentional human transport (Brancatelli & Zalba, 2018). The lack of invasive plant species on ACEP wetlands as compared to other available wetland habitat indicates that ACEP wetlands are contributing native wetland ecosystems to the wetland matrix in the state.

Our findings suggest that wetlands restored on agricultural land through ACEP are comparable to other available wetland habitat in West Virginia in some aspects, while providing different vegetative structure and flooding regimes. We did not find differences between sites in terms of possible stressors from surrounding land cover. ACEP appears to be providing valuable wetland habitat within agricultural landscapes of West Virginia. Most of the differences we observed were due to different vegetative communities between ACEP and reference wetlands, which may contribute to a diversity of wetland ecosystems that could promote wetland biodiversity on a state‐wide scale. Generally, wetland creation or restoration on agricultural land increases regional biodiversity (Thiere et al., 2009), and heterogeneity within agricultural landscapes could combat biodiversity losses associated with agricultural intensification (Benton, Vickery, & Wilson, 2003). Our project highlights the importance of continuing and expanding ACEP in West Virginia.

## CONFLICT OF INTERESTS

The authors have declared that no competing interests exist.

## AUTHOR CONTRIBUTIONS

KEL conceptualized and involved in data curation, formal analysis, investigation, methodology, visualization, writing—original draft preparation, writing—review and editing, and final approval of version to be published. CTR involved in conceptualization, data curation, formal analysis, methodology, project administration, resources, software, supervision, visualization, writing—review and editing, and final approval of version to be published. JTA involved in conceptualization, funding acquisition, methodology, project administration, resources, supervision, writing—review and editing, and final approval of version to be published.

### Open Research Badges

This article has earned an Open Data Badge for making publicly available the digitally‐shareable data necessary to reproduce the reported results. The data is available at https://datadryad.org/stash/share/S75_9JtJfU6ASpt49_sOVE9Db-SW0vAGbBQsgtx90pw and DOI upon data publication: https://doi.org/10.5061/dryad.cjsxksn2b.

## Data Availability

Data is archived in the Dryad Data Repository. Link to data during review process: https://datadryad.org/stash/share/S75_9JtJfU6ASpt49_sOVE9Db-SW0vAGbBQsgtx90pw. DOI upon data publication: https://doi.org/10.5061/dryad.cjsxksn2b.
