# Supplementary material for: A comparison of wetland characteristics between Agricultural Conservation Easement Program and public lands wetlands in West Virginia, USA
Source: Ecol Evol. 2020 Feb 20;10(6):3017–31. doi: 10.1002/ece3.6118 (PMC7083671; doi:10.1002/ece3.6118)
Supplement: Supplementary file 1 [file ECE3-10-3017-s001.docx]

**Table S1.** Agricultural Conservation Easement Program (ACEP) wetlands and reference wetlands located in West Virginia, USA. Table includes description of wetland size, county, physiographic region, wetland class, and number of point counts of each site. For ACEP sites, year of restoration is also included.

| Site | Wetland Size (ha) | County | Physiographic Region | Wetland Class | Year restored (ACEP) |
| --- | --- | --- | --- | --- | --- |
| ACEP 1 | 4.2 | Summers | Appalachian Plateau | forested/scrub-shrub | 2010 |
| ACEP 2 | 1.3 | Greenbriar | Appalachian Plateau | palustrine emergent | 2010 |
| ACEP 3 | 3.2 | Greenbriar | Appalachian Plateau | forested / palustrine emergent | 2012 |
| ACEP 4 | 1.8 | Upshur | Appalachian Plateau | palustrine emergent | 1998 |
| ACEP 5 | 18.6 | Nicholas | Appalachian Plateau | scrub-shrub | 1998 |
| ACEP 6 | 28.7 | Mason | Appalachian Plateau | palustrine emergent | 1996 |
| ACEP 7 | 27.9 | Nicholas | Appalachian Plateau | forested | 1999 |
| ACEP 8 | 1.4 | Clay | Appalachian Plateau | palustrine emergent | 2001 |
| ACEP 9 | 6.0 | Pocahontas | Appalachian Plateau | scrub-shrub | 1998 |
| ACEP 10 | 0.91 | Grant | Alleghany Mountain | palustrine emergent | 2011 |
| ACEP 11 | 3.7 | Upshur | Appalachian Plateau | palustrine emergent | 1999 |
| ACEP 12 | 4.2 | Preston | Alleghany Mountain | palustrine emergent | 1998 |
| ACEP 13 | 3.8 | Taylor | Appalachian Plateau | palustrine emergent | 1998 |
| Reference 1 | 4.5 | Webster | Appalachian Plateau | forested/scrub shrub |  |
| Reference 2 | 7.2 | Monongalia | Appalachian Plateau | palustrine emergent |  |
| Reference 3 | 0.28 | Webster | Appalachian Plateau | palustrine emergent |  |
| Reference 4 | 9.7 | Mason | Appalachian Plateau | palustrine emergent |  |
| Reference 5 | 3.4 | Randolph | Alleghany Mountain | Palustrine emergent |  |
| Reference 6 | 14.3 | Tucker | Alleghany Mountain | palustrine emergent/forested/scrub-shrub |  |
| Reference 7 | 0.95 | Upshur | Appalachian Plateau | palustrine emergent |  |
| Reference 8 | 7.5 | Preston | Alleghany Mountain | palustrine emergent |  |
| Reference 9 | 2.9 | Barbour | Appalachian Plateau | forested/scrub-shrub |  |
| Reference 10 | 6.8 | Barbour | Appalachian Plateau | forested/scrub -shrub |  |
